# Assumptions, Perceptions, and Experiences of Behavioral Health Providers Using Telemedicine: Qualitative Study

**DOI:** 10.2196/48232

**Published:** 2023-10-03

**Authors:** Marcy Ainslie, Marguerite Corvini, Jennifer Chadbourne

**Affiliations:** 1 Department of Nursing University of New Hampshire Durham, NH United States; 2 Telepractice Center University of New Hampshire Durham, NH United States; 3 Department of Agriculture, Nutrition, and Food Systems University of New Hampshire Durham, NH United States

**Keywords:** telemedicine, behavioral health, implementation research, implementation, adoption, telepsychiatry, mental health, psychiatry, clinicians, providers, telehealth, provider, clinician, integration, recommendations, recommendation, guidelines, guideline, perspective, experience

## Abstract

**Background:**

The urgent and reactive implementation of telemedicine during the pandemic does not represent a long-term, strategic, and proactive approach to optimizing this technology. The assumptions, perceptions, and experiences of the behavioral health providers using telemedicine can inform system-wide and institutional-level strategies to promote longitudinal maintenance of care delivery, which can reduce the use of high-cost care due to new symptom onset and symptom exacerbation related to service interruptions.

**Objective:**

We aim to identify the assumptions, perspectives, and experiences of behavioral health clinicians and providers using telemedicine to inform the development of an optimized, sustainable approach to telemedicine implementation.

**Methods:**

This qualitative study applies the domains of the Consolidated Framework for Implementation Research (CFIR) to structure data collection and analysis from behavioral health providers using telemedicine via an audiovisual connection in the New England region. In total, 12 providers across levels of care were recruited for a 60-minute interview, developed from the CFIR interview guide. Atlas Ti Qualitative Software (version 23; ATLAS.ti Scientific Software Development GmbH) was used to coordinate and facilitate coding among 3 reviewers. Deductive coding was provided from the CFIR interview guide, allowing for data to be categorized by domain and construct. Constructs were analyzed for descriptive themes and tabulated for response frequency. Uncoded data were reviewed and coded in vivo to explore variables contributing to participant perceptions of experience with telemedicine use. Descriptive themes, then analytical themes, were identified. Analytical themes and tabulated frequency of response data were summarized. Finally, a sentiment analysis was completed to derive tone and meaning from the data.

**Results:**

Results are reported within the CFIR domains: intervention characteristic, outer setting, inner setting, characteristics of individuals, and process. The findings with ≥90% agreement include “best practice standards were not known”; “telemedicine was believed to be efficient and time-saving for the patient and provider, maximizing productivity and thus increasing access to care”; “telemedicine provided an additional option for patients to access services, promoting sustained continuity and timeliness of care”; “participants did not identify any clear goals related to telemedicine use”; “demonstrated positive affective responses to telemedicine use”; “expressed high efficacy with telemedicine utilization”; and “strong leadership support.”

**Conclusions:**

These findings support the development of interstate compacts advancing licensure across state lines; payment parity across modalities of care to ensure the financial vitality of behavioral health services; improved dissemination of telehealth training and resources, and telehealth training in academic programs of the health professions; seamless, dynamic workflows to accommodate the changing needs of patient and care continuity; emergency response protocols; and community partnerships to provide private spaces needed for a therapeutic encounter. Future research exploring the patient’s experience with telemedicine is needed for all stakeholders to be represented in developing a sustainable, integrated system.

## Introduction

### Overview

After a nationwide reactive approach to telemedicine implementation, this qualitative research explores the shifting assumptions, perceptions, and experiences of behavioral health care clinicians and providers to inform a long-term, sustainable model for telemedicine integration for behavioral health services. Prior to the pandemic, the uptake of telemedicine as a care delivery modality lagged in comparison to technological advances due to regulatory, reimbursement, and systemic stasis. However, the pandemic obliterated barriers to telemedicine uptake as in-person services became severely limited due to closure of outpatient and nonemergent care clinics; health systems proved change was possible. Even so, this context of urgent implementation during a state of emergency does not represent the conditions of our postpandemic health care system. Implementation research that reflects our current health care environment is needed to proactively optimize telemedicine integration.

### Background

About 46.6 million American adults experience mental illness [1]. Pharmacotherapy and behavioral interventions for behavioral illness are effective and widely adopted; however, people with behavioral illness are vulnerable to exacerbations and relapses when there are interruptions in the delivery of care. These interruptions in services often lead to worsening symptoms, or onset of new symptoms, which shifts care from low- to high-cost settings, and results in high financial, personal, and social costs. Continuity of care through effective patient engagement is prioritized in behavioral health care plans. Telemedicine is a modality to deliver behavioral health services that was underused until the pandemic. Optimizing the integration of this technology into care delivery planning may improve continuity of care and patient engagement in maintenance services in the long-term.

In the wake of the pandemic, federal and state governing entities, including Centers for Medicare and Medicaid (CMS), relaxed stringent regulatory requirements to facilitate the adoption of telemedicine and maintain continuity of care. Policy and regulatory changes included lessor restrictions on technological devices (eg, the need to be Health Insurance Portability and Accountability Act [HIPAA] compliant) and the geographic location of the patient and provider at the time of the visit. These changes eliminated geographic barriers and the relaxation of state-specific licensure enabled health care professionals to digitally cross states lines to expand service delivery and reach patients in their homes [2]. Further, CMS flexibilities permitted audio-only contact, allowed hundreds of telemedicine services to be reimbursed at a rate no less than in-person services, and provided the opportunity for waiver of copayments for telemedicine services [3]. These CMS flexibilities around the use of telemedicine have been extended until 2024; however, the Consolidated Appropriations Act of 2023 extended many of the Medicare telemedicine flexibilities authorized during the COVID-19 public health emergency [4].

Research about telemedicine utilization and efficacy must be interpreted through the lens of time, specific available technology, user efficacy of technology, and internet connectivity at the time of this study. Through the widespread use of telemedicine during the pandemic, Schriger et al [5] suggests mental health clinicians were largely satisfied with their telemedicine work which aligns with findings from other studies. Clinicians expressed value in the creative and collaborative opportunities realized through web-based therapy as well as increased access for patients. Interestingly, although attendance and retention rates appear higher for telemedicine visits, client engagement via telemedicine is a notable challenge [6]. Research has demonstrated that individualized, shared decision-making between providers and patients about service modality, whether that be in person or via telemedicine, is an effective approach to sustaining patient engagement in care [2]. Studies evaluating behavioral health provider perceptions during the pandemic found that providers noted that telemedicine affects the visit content, for example, what is discussed and the process of how it is discussed in therapy. The difference in visit content can alter patient engagement, retention, and attendance and can result in variable acceptability across providers and clients [5,6]. While accessibility and convenience proved to be facilitators of use, provider buy-in is important for actual use to occur [7].

Appleton et al [8] concluded in a systematic review of facilitators and barriers to optimal implementation of telemedicine in behavioral health care settings that provider satisfaction during a period when in person visits were extremely limited does not likely equate with long term satisfaction, and challenges persisted over time and became more severe. Connolly et al [9] found that high clinical adoption sites within the Department of Veterans Affairs correlated to the complexity of technological integration and that simple user-friendly processes promote use. Challenges included confidentiality concerns, documentation and scheduling, increased isolation, and fatigue. Benefits included efficiency, improved accessibility, reduced no-shows, and seeing patients in their own environments [5]. However, Frye et al [10] found provider self-efficacy of telemedicine use expanded rapidly within the first month of use and was more of an anticipated barrier than an actual barrier. Technological challenges related to complexity of integration and functionality served as the greatest actual barrier to service delivery by telemedicine [10]. Lipschitz et al [6] found that most providers preferred a hybrid model of care moving forward, with the assumption that reimbursement and licensure barriers are addressed. Aronowitz et al [11] found that the most marginalized populations had the greatest struggle with accessibility due to unstable phone or internet connectivity. Telemedicine cannot replace all in-person visits but instead offers a value-add for certain populations and visit types, though the adoption is likely greatly influenced by perceptions of the health care team.

### Significance

Literature prior to the pandemic does not account for technological advances, policy or regulatory and reimbursement facilitators, and increased adoption that resulted from the pandemic. Literature during the pandemic does not equate to an environment in which telemedicine is optional and not mandated. To implement evidenced based telemedicine practice, illustrating the influences of and by the health care team delivering the service in our current condition is critical.

This research aims to (1) analyze factors influencing health care providers when deciding if, when, for whom, and how telemedicine services are incorporated into a behavioral health setting; (2) inform health care system, institutional, and academic planning; (3) promote policy and regulatory considerations to optimize this care modality in a post pandemic environment.

## Methods

### Research Question

What are the assumptions, perceptions, and experiences of behavioral health providers about synchronous telemedicine delivery of care during the pandemic that are shaping institutional telemedicine practice postpandemic?

### Ethics Approval

The University of New Hampshire institutional review board reviewed the research plan and approved the proposal with exempt status (IRB FY2023-40). Informed consent was passively obtained for engagement in this study from all participants. A hyperlink to the full informed consent document was available through the first page of the Qualtrics survey link with an option to proceed conferring informed consent. The confidentiality and privacy of all participants was ensured by the coding and University System of New Hampshire secure cloud storage of participant contact information, interview video recordings, and associated transcripts. Furthermore, results are reported in the aggregate for the full participant population and by subpopulation of health provider licensure, that is, prescriber versus clinician. No financial or gift compensation was made to participants.

### Participants

Purposive sampling was used to recruit participants who held a license and certification to provide behavioral health services, were actively practicing in a behavioral health setting, and provided at least 4 hours per month of telemedicine services in at least 1 of the past 2 years. Solicitations for participants were made on social media, professional organization websites, and at a regional behavioral health conference. Further, 12 participants met the inclusion criteria, signed the consent form, and completed a 1-hour structured interview on Zoom (Zoom Video Communications, Inc). In total, 5 participants reported working in a practice with >100 employees, 1 worked in an organization with 25 employees, and the remaining 6 were in private practice. Insurance accepted at the practices included only self pay/private insurance in 8 practices, 3 accepted a mix of self pay, private insurance, and Medicare or Medicaid, 1 practice accepted Medicaid only. Practice locations varied with 3 rural practices, 5 urban practices, and 4 in a mixed setting. Participants represented the breadth of licensure supporting mental health services with 5 licensed independent clinical social workers, 4 psychiatric mental health nurse practitioners, and one each of doctor of philosophy in psychiatry, licensed mental health counselor, and licensed mental health therapist. Age of the participants included 1 who was aged >60 years, 8 aged 41-60 years, and 1 aged 25-40 years.

### Theoretical Framework

This research employed the CFIR framework to deductively identify variables impacting clinician and provider adoption and utilization of telemedicine. The validated CFIR interview guide [12] was used to create a structured interview exploring 5 domains: *intervention characteristics* (attributes of telemedicine itself), *outer setting* (characteristics of the external context within which the behavioral health care system functions), *inner setting* (intraorganization characteristics), *individual characteristics* (characteristics of the health care team members using telemedicine), and *process* (implementing telemedicine) to date. Within each domain, key factors that influence the implementation of telemedicine are broken down into multiple constructs. The pandemic mandated the implementation of telemedicine as health care offices were closed. Therefore, questions evaluating the implementation as a pilot test or delivery of care changes as optional ones were discarded. Questions were selected viewed through the lens of a retrospective analysis for those that would create an effective interview structure to be completed in a time of 60 minutes. In total, 23 of the 39 constructs were selected from the CFIR Interview Guide Tool for a semistructured interview (see Multimedia Appendix 1 for the structured interview guide).

### Data Collection

The Institutes of Medicine definition of *telemedicine* was used during the interview to standardize the meaning of the term. “[Telemedicine is] the use of electronic information and communications technologies to provide and support health care when distance separates the participants” [13]. The validated CFIR interview guide was used to create a structured interview. The research team had 1 member interview the participants, generate the transcripts, and label the files with a deidentified naming taxonomy. The participants were queued to not identify by first and last name or place of employment during the recording and the identity was coded in the transcript for participant anonymity to the research team members completing the analysis. This interview was designed to be completed in 60 minutes and contained questions across all 5 implementation domains containing 23 embedded key constructs. First, the domain of the intervention (ie, telemedicine), characteristics, was explored through the constructs of evidence strength and quality, complexity, and design quality and packaging for telemedicine. The second domain explored the outer setting of telemedicine implementation, covering key constructs of relative advantage, patient needs and resources, and external policies and incentives. The third domain explored the inner setting of telemedicine implementation, covering key constructs of structural characteristics, compatibility, goals and feedback, leadership engagement, available resources, access to knowledge and information, networks and communications, culture, implementation climate, and tension for change. Domain 4 explored the characteristics of the clinicians and providers who implemented telemedicine through the constructs of knowledge and beliefs about intervention and self-efficacy. Domain 5 explored the telemedicine implementation process through the key constructs of opinion leaders, external change agents, intervention participants, executing, and reflecting and evaluating. All interviews were conducted over Zoom and recorded. Artificial intelligence was used to draft a cursory copy of the interview transcript, which was then reviewed and edited by the interviewer to develop a finalized intelligent verbatim transcript of the interview that was used for analysis.

### Data Analysis

The CFIR Interview Guide Tool provided standardized questions for each of the domains. The data collected from these questions were coded to the domain from which the question originated. The guide provided definitions for each domain and construct that was applied in data coding. Atlas Ti Qualitative Software (version 23; ATLAS.ti Scientific Software Development GmbH) was used to coordinate and facilitate coding among 3 reviewers. Deductive coding from the CFIR interview guide was entered into the software and responses from all participants were collated under each established code, categorizing the data by domain and construct. Constructs were then analyzed for descriptive themes and tabulated for frequency of responses.

Tabulation tables are automated in the software. Each construct was analyzed independently by 2 reviewers and the results were then compared for interrater reliability. Uncoded data were reviewed and coded in vivo to explore variables contributing to behavioral health professional perceptions and influences of telemedicine implementation. In vivo coding was analyzed for descriptive themes from which analytical themes were identified. Analytical themes and tabulated frequency of response data were then summarized. Finally, a sentiment analysis was completed to derive tone and deeper meaning from the data.

## Results

The results are reported according to the CFIR domains: *intervention characteristic; outer setting; inner setting; characteristics of individuals;* and *process.* Tables 1-3 are categorized by domain and construct. The tables are set up with deductive coding as recommended by the CFIR guidelines. The results reported were tabulated for frequency of responses. As a result of the analytical analysis, the following themes were identified: key policy, regulatory, and environmental considerations; training and education; fidelity of therapeutic environment; emergency response; and continuity of care. Finally, the sentiment analysis revealed an overall positive emotional response to the experience of using telemedicine. The authors’ analysis of the collected data is given in Tables 4 and 5.

**Table 1:** Intervention characteristics that may impact implementation success.

Constructs in this domain	Results	Example quotes
Evidence strength and quality^a^	In total, 92% were not aware of best practice standards. Only 1 had done a formal training prior to the pandemic. Further, 33% reached out to a professional organization or self-solicited. Note, 50% sited anecdotal evidence as to the effectiveness of using telemedicine. Furthermore, 50% were aware that leadership in their respective organizations were in favor of telemedicine. Motivations include financial security and sustaining continuity of patient care.	“I have seen it work” or “my colleague told me it worked for her.”
Relative advantage^b^	In total, 92% recognized the efficiency and time-saving benefit to both the patient and the provider (eg, lack of necessary transit time including parking and preparation of physical appearance). Linked with time management is increased access to care. Fluidity of care delivery: care is not missed because of poor weather, illness, broken car, or any unplanned event that would result in a cancelation. Flexibility of choice: 1 provider reported a patient stating that the privacy of telemedicine helped her seek needed care as the stigma of needing mental health support would have precluded her from going to a public office space. Observing the client’s environment.	Client benefits A participant noted how clients can “work from home, and they just pop off, we do therapy, they pop back on and they make the hour on one end or the other.” “Put the child on for an hour of telemedicine when they may not have the time or additional childcare needed to bring the patient for an in-person visit.” Provider benefits Extended hours, able to fill provider gaps, expanded catchment areas across state lines “Normally they would have had to find another provider and start over, or not and drop out of care; but, with telemedicine we could keep things going.” “Telemedicine removed geographic limitations in finding a provider.” “This means that more clinical care is being given to the general population and more need is being met.” “For the last few years she has been sleeping in her living room, in the den, and so via telemedicine, we’re able to meet and she’s able to do some exposure and some work by going into her bedroom still connected via telemedicine. That you know in the office we would have talked about her practicing, but I probably wouldn’t have been able to be there and kind of coach her through it.”
Complexity^c^	Little complexity for providers delivering telemedicine using the existing technology. Further, 3 participants describe challenges with the older adult population and less with adolescents and young adults, but challenges are improved.	N/A^d^

^a^Evidence strength and quality is the degree to which the practice of telemedicine has robust evidence supporting its effectiveness.

^b^Relative advantage is the degree to which telemedicine as a care delivery mechanism is better than current practice.

^c^Complexity identifies to what extent the intervention is complicated, in scope or process, or disruptive to current practice.

^d^N/A: not applicable.

**Table 2:** Outer setting: the economic, political, and social context that surrounds an organization.

Constructs in this domain	Results	Example quotes
Patient needs and resources^a^	In total, 100% of participants discussed a strong need for patients to have greater options of how to access services to sustain continuity and timeliness of care. Accessibility considerations beyond the pandemic landscape were also discussed regarding opportunities to reach a broader patient population and ease burdens associated with in-person care (eg, cost savings). Further, 75% of participants highlighted barriers such as privacy concerns, the missed opportunities often provided with in-person visits, and lack of broadband access.	“People that are struggling financially, it was a godsend for them because they didn’t have to spend that money driving to see me. I mean, financially, I think it’s way more feasible for the client.” “I had somebody walking along the sidewalk talking to me because that’s the most privacy he could get.” “The sacredness of the therapy room and of the other separateness of ‘I’m going to go to therapy’ and be in that place. And then I’m going to leave that place.” “I’ve had people with terrible cell phone service, so they were really struggling talking and I was like, well, this is just so nontherapeutic.”
Peer pressure^b^	External pressure to implement telemedicine services, telemedicine as a draw to hire quality providers.	“Research from an EAP [employee assistance program] that I work with who highly benefits from their therapist’s offering telemedicine.” “You’ve got all these companies around the country doing telemedicine’ before asking the question ‘Well, why not here?’”
External policies and incentives^c^	In total, 83% of participants mentioned the importance of payment parity and between in-person and telemedicine services. Further, 75% of participants reference challenges to state licensure as a major barrier to providing access to telemedicine across state lines.	“There are some insurance companies that don’t pay telemedicine- don’t pay as much for telemedicine, and so it would be great to see nationwide legislation that says that there’s parity between in-person and telemedicine.” “It would be a different story if it was being paid at half the rate.” “If insurance hadn’t reimbursed for it, and wasn’t reimbursing for it, I wouldn’t consider it. For most people it would be much fewer people that would receive the service.” “It was a very good fit in terms of the type of issue he was struggling with. However, he would split his time between another state and New Hampshire, and it was a split like he would go down there for a couple of weeks, and then come here for a couple of weeks. And because of that, I couldn’t serve him. Because my license restricts me to seeing somebody who is physically sitting in New Hampshire, right? So in that way I don’t think the state licensing boards have stayed up to date or are really being proactive in terms of the way telemedicine has changed the delivery of services.”

^a^Patient needs and resources identifies the degree to which patient needs and barriers are identified and prioritized.

^b^Peer pressure identifies the pressure applied by an outside entity to maintain or establish a competitive advantage or to mimic services offered.

^c^External policies and incentives refers to a broad array of external strategies that drive intervention use including policy and regulations, formal guidelines, and more.

**Table 3:** Inner setting: the places where the intervention is being implemented.

Constructs in this domain	Results	Example quotes
Access to information and technology^a^	Accessibility considerations beyond the pandemic landscape were also discussed regarding opportunities to reach a broader patient population and ease burdens associated with in-person care (eg, cost savings). Further, 75% of participants highlighted barriers such as privacy concerns, the missed opportunities often provided with in-person visits, and lack of broadband access.	“People that are struggling financially, it was a godsend for them because they didn’t have to spend that money driving to see me. I mean, financially, I think it’s way more feasible for the client.” “I had somebody walking along the sidewalk talking to me because that’s the most privacy he could get.” “The sacredness of the therapy room and of the other separateness of ‘I’m going to go to therapy’ and be in that place. And then I’m going to leave that place.” “I’ve had people with terrible cell phone service, so they were really struggling talking and I was like, well, this is just so nontherapeutic.”
Available resources^b^	External pressure to implement telemedicine services, telemedicine as a draw to hire quality providers.	“Research from an EAP [employee assistance program] that I work with who highly benefits from their therapist’s offering telemedicine.” “You’ve got all these companies around the country doing telemedicine’ before asking the question ‘Well, why not here?’”
Compatibility^c^	In total, 83% of participants mentioned the importance of payment parity and between in-person and telemedicine services.	“There are some insurance companies that don’t pay telemedicine- don’t pay as much for telemedicine, and so it would be great to see nationwide legislation that says that there’s parity between in-person and telemedicine.” “It would be a different story if it was being paid at half the rate.” “If insurance hadn’t reimbursed for it, and wasn’t reimbursing for it, I wouldn’t consider it. For most people it would be much fewer people that would receive the service.” “It was a very good fit in terms of the type of issue he was struggling with. However, he would split his time between another state and New Hampshire, and it was a split like he would go down there for a couple of weeks, and then come here for a couple of weeks. And because of that, I couldn’t serve him. Because my license restricts me to seeing somebody who is physically sitting in New Hampshire, right? So in that way I don’t think the state licensing boards have stayed up to date or are really being proactive in terms of the way telemedicine has changed the delivery of services.”
Culture^d^	In total, 58% participants felt that the culture of their organization is fully supportive of telemedicine	N/A^e^
Goals^f^	In total, 92% of respondents did not identify any clear goals	N/A
Leadership engagement^g^	Most participants who made statements around leadership engagement stated that it was overall positive in terms of their involvement and commitment to the process of telemedicine utilization.	“There was the executive director, the executive clinical director, the director of operations, the director of IT heavily involved.” “The community leaders were beyond thrilled because there were minimal gaps in service... [and] we kept the revenue going.”
Implementation climate^h^	In total, 67% stated that their overall implementation climate was positive, but rapid-fire.	“Over the course of one weekend, they implemented these procedures, they delivered laptops to clinicians that needed them...” “Pandemic pressure”; “weekend of cramming”; “necessity is the mother of invention.”
Networks and communication^i^	Most participants that discussed networks and communication were self-identified, professional networks.	“Crowd-sourcing of the therapy world.”
Structural characteristic^j^	Those with a private practice had to modify their physical workspace and rely on their own resources for IT, while those part of a larger system did receive assistance. Further, 7 of the participants made statements indicating that their organization had positive characteristics. 4 participants noted the structural characteristics that hindered growth.	“Our buildings are very old, and we run out of space. I think having enough offices has definitely impacted whether we kind of stay on board with doing a lot of telemedicine or not.”
Tension for change^k^	In total, 83% identified the main tension for change was the need to continue access to care for patients and reduce COVID exposure.	“So I really took a lot of guidance from what was happening, at the state level.... This is the way we have to practice if we’re gonna continue to maintain continuity of care for our clients.” “the company, even though they don’t like it, has been more open and accepting of telemedicine.... So they’ve been forced to swallow that jagged pill.”

^a^Access to information and technology identifies the extent to which there is training and direction to guide employees.

^b^Available resources identify the extent to which an organization has the funding, space, materials, and equipment to adequately support a telemedicine intervention implementation.

^c^Compatibility identifies the extent to which telemedicine intervention fits within existing processes, workflows, and systems.

^d^Culture explores the extent to which the organization’s general beliefs, values, and assumptions support the implementation of a telemedicine intervention.

^e^N/A: not applicable.

^f^Goals refers to the goals identified for implementation of the telemedicine intervention.

^g^Leadership engagement pertains to the involvement, commitment, and accountability of leaders and managers to the intervention of telemedicine.

^h^Implementation climate explores the extent to which the organization was receptive to implementing a telemedicine intervention.

^i^Networks and communication seek to identify the “go to” people for solving a problem or accomplishing a goal.

^j^Structural characteristics refers to the extent to which employees have the physical, work, and information technology infrastructure in place to support use of telemedicine.

^k^Tension for change identifies the extent to which change needs to occur because the present situation is unsustainable.

**Table 4:** Characteristics of the individual: individuals involved in the intervention.

Constructs in this domain	Results	Example quotes
Knowledge and beliefs about the intervention^a^	In total, 92% demonstrated positive affective response to telemedicine and half shared beliefs about telemedicine with a negative affect. Further, 58% discussed their perception of the intervention’s viability with consideration for factors such as convenience, flexibility, ease of use, access, and quality of care.	“I’m saying it as a barrier, but it could be a really positive thing.” “Some populations do better than others on telemedicine.” “Didn’t feel it was a great fit for children.” “It gives you a lot of flexibility. I love it.” and another stated “it allows access where access wasn’t there… for transportation reasons, for economic reasons.” “Studies that show that it’s very hard to build a rapport with the client via telemedicine. So that would have been a consideration for me, had I not already been doing it. I don’t think that’s the case. I have built rapport easily with clients.”
Self-efficacy^b^	In total, 100% of participants expressed high efficacy with telemedicine use.	”It’s easier to do telemedicine than it is doing in person work.” “I’m very confident. Initially I wasn’t.” “It’s very easy for me, very easy. Not complicated at all.”

^a^Access knowledge and beliefs about the intervention identifies attitudes toward, and values placed on, the intervention by individuals as well as their general knowledge about the intervention.

^b^Self-efficacy identifies an individual’s confidence in their ability to achieve goals through effective action.

**Table 5:** Process domain: stages of implementation and key stakeholders.

Constructs in this domain	Results	Example quotes
Engaging^a^	In total, 92% reported strong leadership support of telemedicine during COVID. As COVID restrictions rolled back, it is unclear who the champions of telemedicine are. The majority of providers favored a hybrid approach, where telemedicine and in person visits were both options and could be decided in a shared decision-making approach, based on a multitude of variables.	“Providers are more receptive with telemedicine when treating a patient than they are of a zoom meeting that we sit in every hour. Finally, someone said, ‘we’re chucking these out the window because we can’t take it anymore- we want a meeting, put us together.’”
Executing^b^	In total, 100% of participants expressed high efficacy with telemedicine use. In total, 3 independent business owners noted transitional support among colleagues in developing a plan. Further, 5 of the participants described adaptations to their plans based on patient needs.	“Everything went seamlessly.” “I do tend to start over the phone because I think that’s where most people are most comfortable. There’s a lot less technology issues and I can explain our security features to reduce any anxiety about those things.”
Reflecting and evaluating^c^	In total, 1 out of the 12 participants noted there was a systematic evaluation of telemedicine utilization and satisfaction survey completed by patients. None of the participants were aware of any established outcome measures that were identified or tracked to inform system improvements. Further, 67% attest to informally asking the patient if telemedicine is working for them and looking at informal measures such as client retention and client preference.	“You are the first person to ever ask me what I thought about telemedicine or how it is working for me.”

^a^Engaging considers to what extent option leaders, champions, external change agents, and intervention participants were present to impact the success of the implementation.

^b^Executing considers to what extent to which the intervention was carried out or accomplished according to a plan.

^c^Reflecting and evaluating identifies the degree to which the telemedicine implementation was successful.

## Discussion

### Principal Findings

This work confirms key findings of prior studies while adding a nuanced, contextual layer of internal and external organizational structure and environmental impacts influencing the acceptability and utilization of telemedicine by behavioral health providers. Similar to findings by Downing [14] and Cowan et al [15], this study found that most providers did not receive formal telemedicine training in their degree programs, which exposed a gap in knowledge and experience for many practicing providers during the pandemic. Despite the availability of many evidence-based resources to support telemedicine implementation from sources such as the Center for Telemedicine Innovation, Education, and Research [16], the Center for Connected Health Policy [17], the CMS, and the Health Resources and Services Administration [18], there is a lack of provider awareness, effective dissemination, and uptake of telemedicine training tools.

This study also confirmed prior findings [15,19] that administrative, regulatory, and licensure barriers often slow effective implementation and use of telemedicine in behavioral health care services. First, removal of prepandemic regulatory practices around interstate licensure and practice made telemedicine adoption feasible in areas of need without adding an additional administrative burden on the already strained workforce. The educational preparation of our mental health providers is accredited and regulated at a national level, thus licensure at the state level creates unnecessary barriers to care delivery. Removing state level licensure requirements improved access to care by mitigating the provider shortages that result from the known irregular distribution of behavioral health providers across the nation. Second, reimbursement of telemedicine visits at the same rate as in-person rates promoted the financial viability of the practice and the personal protection from viral spread during an epidemic that was needed to make the delivery of behavioral health services through telemedicine possible. This research suggests that a lack of long-term commitment to payment parity will put telemedicine services at risk of being canceled.

Results from the inner setting domain highlight the need for a seamless workflow and resources that facilitate a multimodal care delivery model. Specifically, issues of privacy, emergency response, and evaluation were highlighted. First, a common barrier to both patients and providers was a lack of private, uninterrupted space to advance the fidelity of a therapeutic environment. Health care organizations may consider partnering with existing community-based facilities, such as libraries, town halls, and event spaces, for private rooms that are Wi-Fi enabled and may be accessed by both patients and providers. Second, the need for an emergency preparedness plan that is tailored to the nuances of a behavioral health telemedicine visit, specifically suicidality, is needed. During an in-person visit, a person deemed at risk to themselves, or others is held to establish a safety plan, or to provide supervision, when indicated. The physical space present between participants during a behavioral health telemedicine visit was of great concern among providers. In 2022, the 211 emergency behavioral health phone helpline went into effect nationally in response to this identified need. Lastly, the process domain highlighted a key area for organizational improvement in evaluation. A rapid cycle quality improvement plan was lacking during the pandemic and desired by most research participants. Smart goals and metrics to evaluate telemedicine outcomes, fidelity, and ongoing process improvement are likely to improve the quality of care delivered.

Continuity of mental health services is critical to prevent the exacerbation or onset of new symptoms, which leads to evaluations and stabilizations that are costly, both socially and financially. In lieu of continuing the debate about the superiority of in-person versus telemedicine visits, acknowledging the clinical reality of prioritizing continuity of high-quality care using all means available may help to develop dynamic health systems that can respond to the changing needs of the patient. Individualized and dynamic care plans that use the most appropriate care delivery modality for a single point in time, whether in-person or telemedicine, would require both modalities to be available to all providers and patients. While these findings echo recommendations previously noted by Lipschitz et al [6], this work emphasizes the need for continual reassessment of the most supportive care delivery modality to recognize that the therapeutic needs of the patient change rapidly due to many nonclinical and clinical environmental and social factors. Efforts toward continual re-evaluation of what is the most appropriate care delivery modality may lead to improved engagement with maintenance services, avoiding the high costs associated with emergency services.

In order to realize this ideal of a dynamic system, findings from this study suggest key policy, regulatory, and environmental considerations critical to a successful, long-term integration of telemedicine services in our postpandemic health care system. The key findings from this research suggest a need for interstate licensure compacts; payment parity for all modalities of care to ensure the financial vitality of behavioral health services, a historically underfunded area of service; improved dissemination of telemedicine training and utilization resources; telemedicine curriculum in academic programs of the health professions; to develop seamless dynamic workflows to accommodate the changing needs of the patient and promote continuity of care; to develop emergency response protocols; and to develop community partnerships to provide private spaces needed for therapeutic encounters.

### Limitations and Areas for Future Research

This study was conducted in a rapidly changing landscape and presents 1 snapshot in time of current practice. Additionally, the sample size was limited to 12 participants. It is a demographic and geographic representational limitation that all 12 participants were behavioral health providers located in New England, although the representation across different levels of care available in behavioral health is an asset. An additional limitation is the lack of representation of those in leadership positions. While this study focused on those operationalizing telemedicine practice in direct patient care, the leadership perspective may have provided additional insights. While there is good representation of diverse types of organizations, future studies would benefit by focusing on the specific needs within organizational types. Moreover, the rapid widespread use of telemedicine allowed for neither an in-depth study of potential access and utilization concerns across populations and socioeconomic status, nor an empirical examination of the efficacy of this modality for care of people with serious mental illness, specifically schizophrenia and bipolar disorder. Research informing the validity and efficacy of telemedicine interventions from the perspective of the patient is needed to inform implementation, policy, and future regulatory directions.

### Conclusions

The implementation of telemedicine was urgently operationalized to mitigate the spread of COVID-19 while maintaining continuity of care during the pandemic. Research into the assumptions, perceptions, and experiences of behavioral health providers using telemedicine during this time can support health system and organizational planning for long-term telemedicine integration into the delivery of health care services. Findings from this study add to the growing body of literature that is emerging from lessons learned during the pandemic and offer policy, regulatory, environmental, and organizational consideration to support a dynamic delivery of care model that better meets the changing needs of the patients.

## Appendix

Multimedia Appendix 1 Consolidated Framework for Implementation Research (CFIR) structured interview guide selection.

## Abbreviations

CFIR: Consolidated Framework for Implementation Research
CMS: Centers for Medicare and Medicaid
HIPAA: Health Insurance Portability and Accountability Act

## Data Availability

The data sets generated and analyzed during this study are available from the corresponding author on reasonable request.

## References

[1] (2023). Mental illness. National Institute of Mental Health.

[2] Ainslie M, Brunette MF, Capozzoli M (2022). Treatment interruptions and telemedicine utilization in serious mental illness: retrospective longitudinal claims analysis. JMIR Ment Health.

[3] (2023). Coronavirus waivers and flexibilities. Centers for Medicare and Medicaid Services.

[4] (2023). Consolidated Appropriations Act: In the senate of the United States—117th Cong., 2d Sess. H.R. 2617. US Senate Committee on Appropriations.

[5] Schriger SH, Klein MR, Last BS, Fernandez-Marcote S, Dallard N, Jones B, Beidas RS (2022). Community mental health clinicians' perspectives on telehealth during the COVID-19 pandemic: mixed methods study. JMIR Pediatr Parent.

[6] Lipschitz JM, Van Boxtel R, Torous J, Firth J, Lebovitz JG, Burdick KE, Hogan TP (2022). Digital mental health interventions for depression: scoping review of user engagement. J Med Internet Res.

[7] Galvin E, Desselle S, Gavin B, Quigley E, Flear M, Kilbride K, McNicholas F, Cullinan S, Hayden J (2022). Patient and provider perspectives of the implementation of remote consultations for community-dwelling people with mental health conditions: a systematic mixed studies review. J Psychiatr Res.

[8] Appleton R, Williams J, San Juan NV, Needle JJ, Schlief M, Jordan H, Rains LS, Goulding L, Badhan M, Roxburgh E, Barnett P, Spyridonidis S, Tomaskova M, Mo J, Harju-Seppänen J, Haime Z, Casetta C, Papamichail A, Lloyd-Evans B, Simpson A, Sevdalis N, Gaughran F, Johnson S (2021). Implementation, adoption, and perceptions of telemental health during the COVID-19 pandemic: systematic review. J Med Internet Res.

[9] Connolly SL, Sullivan JL, Lindsay JA, Shimada SL, Heyworth L, Weaver KR, Miller CJ (2022). Factors influencing uptake of telemental health via videoconferencing at high and low adoption sites within the Department of Veterans Affairs during COVID-19: a qualitative study. Implement Sci Commun.

[10] Frye WS, Gardner L, Campbell JM, Katzenstein JM (2022). Implementation of telehealth during COVID-19: implications for providing behavioral health services to pediatric patients. J Child Health Care.

[11] Aronowitz SV, Engel-Rebitzer E, Dolan A, Oyekanmi K, Mandell D, Meisel Z, South E, Lowenstein M (2021). Telehealth for opioid use disorder treatment in low-barrier clinic settings: an exploration of clinician and staff perspectives. Harm Reduct J.

[12] Damschroder LJ, Aron DC, Keith RE, Kirsh SR, Alexander JA, Lowery JC (2009). Fostering implementation of health services research findings into practice: a consolidated framework for advancing implementation science. Implement Sci.

[13] Field MJ, Institute of Medicine (US) Committee on Evaluating Clinical Applications of Telemedicine (1996). Introduction and background. Telemedicine: A Guide to Assessing Telecommunications for Health Care.

[14] Downing J (2021). Assessing nurse practitioner knowledge, confidence, and attitudes in using telemedicine in primary care [dissertation]. University of Kansas.

[15] Cowan KE, McKean AJ, Gentry MT, Hilty DM (2019). Barriers to use of telepsychiatry: clinicians as gatekeepers. Mayo Clin Proc.

[16] (2023). Center for Telemedicine, Innovation, Education, and Research (C-TIER).

[17] Center for Connected Health Policy.

[18] Tips for Telemedicine Success. U.S. Department of Health and Human Services.

[19] Brooks E, Turvey C, Augusterfer EF (2013). Provider barriers to telemental health: obstacles overcome, obstacles remaining. Telemed e-Health.

