# Supplementary material for: Assumptions, Perceptions, and Experiences of Behavioral Health Providers Using Telemedicine: Qualitative Study
Source: JMIR Form Res. 2023 Oct 3;7:e48232. doi: 10.2196/48232 (PMC10582816; doi:10.2196/48232)
Supplement: Multimedia Appendix 1 [file formative_v7i1e48232_app1.docx]

APPENDIX A

CFIR Structured Interview Guide Selection

Intervention Characteristics

**Evidence Strength & Quality**

1. What kind of information or evidence are you aware of that shows whether or not the intervention will work in your setting?
   - What evidence have you heard about from your own research? Practice guidelines? Published literature? Co-workers? Other settings?
   - How does this knowledge affect your perception of the intervention?
2. *In a healthcare setting, influential stakeholders may include influential and well-respected clinicians, where as in an education setting, this may include influential and well-respected teachers or educators.*

What do influential stakeholders think of the intervention?

- - What do administrative or other leaders think of the intervention?

**Relative Advantage**

1. How does the intervention compare to other alternatives that may have been considered or that you know about?
   - What advantages does the intervention have compared to these other programs?
   - What disadvantages does the intervention have compared to these other programs?

**Complexity**

1. How complicated is the intervention?
   - Please consider the following aspects of the intervention: duration, scope, intricacy and number of steps involved and whether the intervention reflects a clear departure from previous practices.

**Design Quality & Packaging**

1. What supports, such as online resources, marketing materials, or a toolkit, are available to help you implement and use the intervention?
   - How do you access these materials?

Outer Setting

**Patient Needs & Resources**

1. To what extent is staff aware of the needs and preferences of the individuals being served by your organization?
2. How well do you think the intervention will meet the needs of the individuals served by your organization?
   - In what ways will the intervention meet their needs? E.g. improved access to services? Reduced wait times? Help with self-management? Reduced travel time and expense?
3. How do you think the individuals served by your organization will respond to the intervention?
4. What barriers will the individuals served by your organization face to participating in the intervention?
5. Have you elicited information from participants regarding their experiences with the intervention?
   - What are their perceptions of the intervention?
   - Can you describe what kind of specific information you have heard?
6. Have you heard stories about the experiences of participants with the intervention?
   - Can you describe a specific story?

**External Policies & Incentives**

1. What kind of financial or other incentives influenced the decision to implement the intervention?
   - How will the intervention affect your organization's ability to receive these incentives?
   - How will the new intervention affect payment or revenue for your organization?

Inner Setting

**Structural Characteristics**

1. How will the infrastructure of your organization (social architecture, age, maturity, size, or physical layout) affect the implementation of the intervention?
   - How will the infrastructure facilitate/hinder implementation of the intervention?
   - How will you work around structural challenges?
2. What kinds of infrastructure changes will be needed to accommodate the intervention?
   - Changes in scope of practice? Changes in formal policies? Changes in information systems or electronic records systems? Other?
   - What kind of approvals will be needed? Who will need to be involved?
   - Can you describe the process that will be needed to make these changes?

**Networks & Communications**

1. When you need to get something done or to solve a problem, who are your "go-to" people?
   - Can you describe a recent example?

**Culture**

1. How would you describe the culture of your organization? Of your own setting or unit?
   - Do you feel like the culture of your own unit is different from the overall organization? In what ways?
2. How do you think your organization's culture (general beliefs, values, assumptions that people embrace) will affect the implementation of the intervention?
   - Can you describe an example that highlights this?

**Implementation Climate**

1. What is the general level of receptivity in your organization to implementing the intervention?
   - Why?

**Tension for Change**

1. Is there a strong need for this intervention?
   - Why or why not?
   - Do others see a need for the intervention?
2. How do people feel about current programs/practices/process that are available related to the intervention?
   - To what extent do current programs fail to meet existing needs? Will the intervention meet these needs?
   - How will the intervention fill current gaps?

**Compatibility**

1. How well does the intervention fit with existing work processes and practices in your setting?
   - What are likely issues or complications that may arise?

**Goals & Feedback**

1. Have you/your unit/your organization set goals related to the implementation of the intervention?
   - [If yes] What are the goals?
2. How does implementation of the intervention align with other organizational goals?

**Readiness for Implementation**

**Leadership Engagement**

1. What kind of support or actions can you expect from leaders in your organization to help make implementation successful?
   - Who are these leaders? How do attitudes of different leaders vary?
   - What kind of support can you expect going forward? Can you provide specific examples?
   - What types of barriers might they create?

**Available Resources**

1. Do you expect to have sufficient resources to implement and administer the intervention?
   - [If Yes] What resources are you counting on? Are there any other resources that you received, or would have liked to receive?
   - What resources will be easy to procure?
   - [If no] What resources will not be available?
2. How do you expect to procure necessary resources?
   - Who will be involved in helping you get what is needed?
   - What challenges do you expect to encounter?

**Access to Knowledge & Information**

1. What kind of training is planned for you? For colleagues?
   - Do you feel the training will prepare you to carry out the roles and responsibilities expected of you? Can you explain?
   - What are the positive aspects of planned training?
   - What is missing?
   - What kind of continued training is planned?
2. What kinds of information and materials about the intervention have already been made available to you?
   - Copies of materials?
   - Personal contact?
   - Internal information sharing; e.g., staff meetings?
   - Has it been timely? Relevant? Sufficient?
3. Who do you ask if you have questions about the intervention or its implementation?
   - How available are these individuals?

Characteristics of Individuals

**Knowledge & Beliefs about the Intervention**

1. How do you feel about the intervention being used in your setting?
   - How do you feel about the plan to implement the intervention in your setting?
   - Do you have any feelings of anticipation? Stress? Enthusiasm? Why?

**Self-efficacy**

1. How confident are you that you will be able to use the intervention?
   - What gives you that level of confidence (or lack of confidence)?
2. How confident do you think your colleagues feel about using the intervention?
   - What gives them that level of confidence (or lack of confidence)?

Process

**Engaging**

**Opinion Leaders**

1. What are influential individuals saying about the intervention?
   - Who are these influential individuals?
   - To what extent will they influence others' use of the intervention? The success of the implementation?

**External Change Agents**

1. Will someone (or a team) outside your organization be helping you with implementing the intervention?
   - Can you describe this person/group?
   - How did they get involved?
   - What is their role?
   - What kind of activities will they be doing?
   - How helpful do you think he/she/they will be? In what ways?

**Intervention Participants**

1. How will you or your colleagues communicate to the individuals that are served by your organization about the intervention?
   - How will they participate in the intervention?
   - How will they access the intervention?

**Executing**

1. Has the intervention been implemented according to the implementation plan?
   - [If Yes] Can you describe this?
   - [If No] Why not?

**Reflecting & Evaluating**

1. How will you assess progress towards implementation or intervention goals?
   - How will results of the evaluation be distributed to stakeholders?
2. Will feedback be elicited from staff? From the individuals served by your organization?
   - [If yes] What kind of feedback?
